# Analysis of Transmission and Control of Tuberculosis in Mainland China, 2005–2016, Based on the Age-Structure Mathematical Model

**DOI:** 10.3390/ijerph14101192

**Published:** 2017-10-07

**Authors:** Yu Zhao, Mingtao Li, Sanling Yuan

**Affiliations:** 1 School of Public Health and Management, Ningxia Medical University, Yinchuan 750004, China; zhaoyuzy123@163.com; 2 School of Mathematics and Computer Science, Ningxia Normal University, Guyuan 756000, China; 3 School of Computer and Information Technology, Shanxi University, Taiyuan 030006, China; limingtao18@126.com; 4 Complex Systems Research Center, Shanxi University, Taiyuan 030006, China; 5 College of Science, University of Shanghai for Science and Technology, Shanghai 200093, China

**Keywords:** tuberculosis, age group, basic reproduction number, global stability, data fitting, uncertainty and sensitivity analysis

## Abstract

Tuberculosis (TB), an air-borne infectious disease, is a major public-health problem in China. The reported number of the active tuberculosis cases is about one million each year. The morbidity data for 2005–2012 reflect that the difference in morbidity based on age group is significant, thus the role of age-structure on the transmission of TB needs to be further developed. In this work, based on the reported data and the observed morbidity characteristics, we propose a susceptible-exposed-infectious-recovered (SEIR) epidemic model with age groupings, involving three categories: children, the middle-aged, and senior to investigate the role of age on the transmission of tuberculosis in Mainland China from 2005 to 2016. Then, we evaluated the parameters by the Least Square method and simulated the model and it had good alignment with the reported infected TB data in Mainland China. Furthermore, we estimated the basic reproduction number $R_{0}$ of 1.7858, with an obtained 95% confidence interval for $R_{0}$ of (1.7752,1.7963) by Latin hypercube sampling, and we completed a sensitivity analysis of $R_{0}$ in terms of some parameters. Our study demonstrates that diverse age groups have different effects on TB. Two effective measures were found that would help reach the goals of the World Health Organization (WHO) End TB Strategy: an increase in the recovery rate and the reduction in the infectious rate of the senior age group.

## 1. Introduction

Tuberculosis (TB) is an air-borne infective disease caused by the slowly-replicating bacterium Mycobacterium tuberculosis (Mtb). Person-to-person transmission of Mtb occurs via the respiratory system, which can happen through both close contact between people and through infectious bacilli being carried throughout buildings by air currents [1]. According to the World Health Organization’s (WHO) Global Tuberculosis Report 2013 [2], an estimated 8.6 million new cases of TB and 1.3 million deaths, including 320,000 deaths among HIV-positive people, were recorded in 2012. Approximately 80% of all new TB cases in the world occur in 22 high burden countries that have incidence rates from 59 to 1003 per 100,000 people. India and China have the largest number of cases at 26% and 12% of the global total, respectively. Despite widespread implementation of control measures, including the Bacillus Calmette Guerin (BCG) vaccination, antiretroviral therapy, antimicrobial chemotherapy, and the WHO’s short course chemotherapy strategy, TB remains a major cause of illness and death in most high-incidence countries.

**Figure 1:**
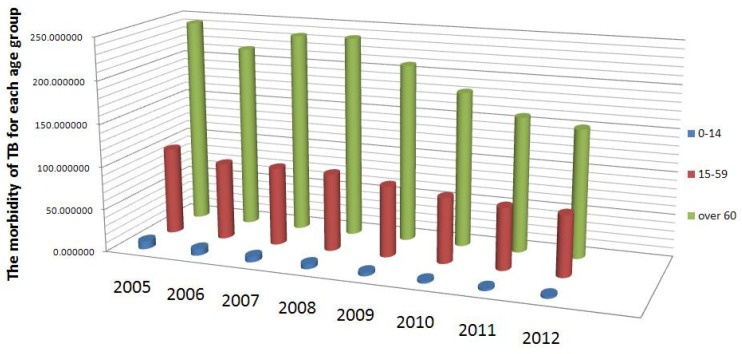
The tuberculosis (TB) morbidity based on age structure from 2005 to 2012 in China (source: CDC, China).

As in many high-TB burden countries, TB is a major public health problem in China. Attributed to the emergence of drug-resistant strains of Mtb and the spread of HIV/AIDS, the TB burden in China has increased over the past two decades [3]. Meanwhile, according to the United Nations, as a result of the one-child policy and low mortality, China is ageing more rapidly than almost any other country [4]. From the morbidity statistics from 2005 to 2012, shown in Figure 1, we know that the difference in morbidity between different age groups is remarkable: the morbidity of the 0–14 age group is the lowest and that of the 60 and older age group is the highest, while the morbidity of the 15–59 age group is in between. However, the role of this age grouping pattern in the transmission of TB in China is not yet clear. Therefore, we wanted to identify the potential effect of age on the morbidity for the transmission of TB in China, which could help provide effective control strategies for diverse sub-populations.

Mathematical modeling has become a powerful tool for analyzing epidemiological characteristics [5,6,7,8,9]. Different models have been developed for defining target sub-populations for treating latent TB infections and incorporating certain factors, such as drug-resistant strains, co-infection with HIV, relapse, re-infection, and vaccination, to study the transmission dynamics of TB [7,10,11,12,13,14,15,16,17,18,19,20,21,22,23,24]. In particular, Blower et al. [10] proposed a simple TB transmission model and presented a theoretical framework for assessing the intrinsic TB transmission dynamics. Bhunuet et al. [14] considered a TB model incorporated the treatment of infectives and chemoprophylaxis. Liu et al. [19] studied a TB model incorporating seasonality. Huynh et al. [24] developed an individual-based computational model to explore the trajectory of the TB burden if the DOTS strategy is maintained or if new interventions are introduced. A more detailed discussion on different TB models was completed by White and Garnett [25]. However, few works have used mathematical models with age groupings to study the transmission of TB in China. In this paper, based on the reported data and the observed morbidity characteristics, we created a susceptible-exposed-infectious-recovered (SEIR) model with age groups of childhood, middle-aged, and senior, to investigate the role of age on the transmission process and evaluate feasible control strategies to reach the goals outlined in the WHO End TB Strategy. We estimated the basic reproduction number $R_{0}$, analyzed the globally dynamic behavior of the model, and used the model to simulate the annual data of infected TB cases reported by the Center for Disease Control (CDC) from 2005 to 2016. Finally, we completed uncertainty and sensitivity analysis of $R_{0}$, and explored some effective and targeted control measures for the transmission of TB in China. The rest of this paper is organized as follows. In Section 2, we present the data collection; formulate the TB model; obtain the theoretical results, such as existence and uniqueness of the solution; and define the basic reproduction ratio $R_{0}$ and global stability of disease-free equilibrium. In Section 3, data fitting and sensitivity analysis of $R_{0}$ are shown, and the feasibility of the WHO End TB Strategy is assessed. A brief discussion ensues in Section 4.

## 2. Materials and Methods

### 2.1. Data Collection

The reported annual and cumulative Tuberculosis cases in Mainland China from 2005 to 2016 were obtained from the National Notifiable Disease Surveillance System (NNDSS) (Table 1). Between 2005 and 2012, a total of 14,221,317 active TB cases were reported. The number of new infected TB cases declined 4.479% per year, from 1,259,308 in 2005 to 836,236 in 2016.

### 2.2. Model Formulation

**Figure 2:**
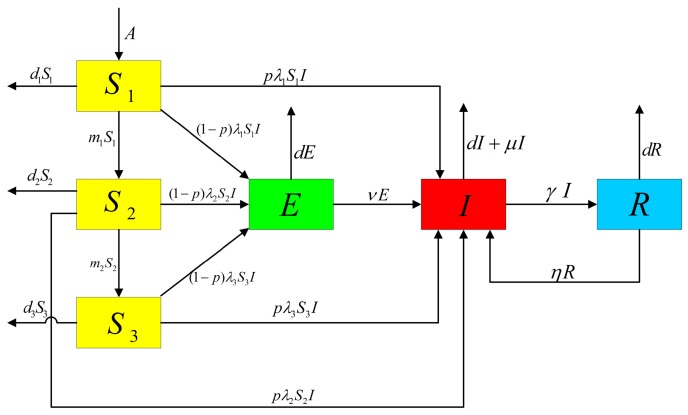
Flow chart of TB transmission with age-structure.

In this section, we introduce a deterministic TB model incorporating age grouping with control measures. The entire population is classified into four classes: susceptible (*S*), latency (*E*), infectious (*I*) and recovered (*R*). Based on the observation that the morbidity among diverse age groups is significantly different (Figure 1), to explore the role of age on the infection pattern between susceptible and infectious classes, the susceptible class was further divided into three age groups: childhood ($S_{1}$), middle-aged ($S_{2}$), and senior ($S_{3}$). We also assumed that the latent, infectious, and recovered classes are the same for different age groups. Since the latent TB cases, which are individuals who have been infected by TB bacteria but are asymptomatic, and cured TB cases may not directly cause death [26], we assumed that the death rate of the latent and recovered classes were related to the natural death rate *d*. Additionally, for infectious class, we added the term μ, based on natural death rate *d*, to describe the deaths caused by TB infection. Our assumptions for the dynamic transmission of TB in China with age groupings are demonstrated in Figure 2.

The model we created has the compartmental structure of the classical SEIR epidemic model, and is described by the following differential equations:

(1) $$\left\{\begin{array}{cc} \frac{dS_{1}}{dt} & =A‒d_{1}S_{1}‒m_{1}S_{1}‒\lambda_{1}S_{1}I,\\ \frac{dS_{2}}{dt} & =m_{1}S_{1}‒d_{2}S_{2}‒\lambda_{2}S_{2}I‒m_{2}S_{2},\\ \frac{dS_{3}}{dt} & =m_{2}S_{2}‒d_{3}S_{3}‒\lambda_{3}S_{3}I,\\ \frac{dE}{dt} & =\left(1‒p\right)\left[\lambda_{1}S_{1}I+\lambda_{2}S_{2}I+\lambda_{3}S_{3}I\right]‒vE‒dE,\\ \frac{dI}{dt} & =p\left(\lambda_{1}S_{1}I+\lambda_{2}S_{2}I+\lambda_{3}S_{3}I\right)‒\left(d+\gamma +\mu \right)I+vE+\eta R,\\ \frac{dR}{dt} & =\gamma I‒\eta R‒dR, \end{array}\right.$$

where all the parameters are positive. *A* is the annual birth rate of the population; $m_{1}$ and $m_{2}$ are the conversion rates from the susceptible children to the susceptible middle-aged group, and from the susceptible middle-aged group to the susceptible senior group, respectively; $\lambda_{1},\lambda_{2}$ and $\lambda_{3}$ are the morbidities of children, middle-aged, and senior susceptible age groups, respectively; *p* is the fraction of fast-developing infectious cases; *v* is the re-activation rate of the latent TB patients; $d_{1},d_{2}$ and $d_{3}$ are, respectively, the mortalities of the adolescent, the middle-aged and the elderly susceptible age groups; *d* is the natural death rate; μ is the disease-induced death rate; γ is the recovery rate; and η is the recurrence rate of successfully treated TB cases.

Due to the severity of the transmission situation, China developed and implemented two five-year national plans in the 1980s and one 10-year national plan in the 1990s to control TB. After implementing these national TB control programs, the modern TB control strategy was implemented. Subsequently, China increased high-quality directly observed treatment, short course chemotherapy (DOTS) [24], and a compulsory Bacillus Calmette Guerin (BCG) immunization program for newborns [27]. These actions helped to effectively control the increase of TB in China. Given this, and based on Model (1), we considered two kinds of control strategies for TB in China: the incremental recovery rate per year due to DOTS, ξ (0<ξ<1), and the immunity rate of the BCG vaccine, φ (0<φ<1). By assuming the newborns that received the BCG vaccine remain in the susceptible compartment, Model (1) becomes the following:

(2) $$\left\{\begin{array}{cc} \frac{dS_{1}}{dt} & =A‒d_{1}S_{1}‒m_{1}S_{1}‒\lambda_{1}S_{1}I,\\ \frac{dS_{2}}{dt} & =m_{1}S_{1}‒d_{2}S_{2}‒\lambda_{2}S_{2}I‒m_{2}S_{2},\\ \frac{dS_{3}}{dt} & =m_{2}S_{2}‒d_{3}S_{3}‒\lambda_{3}S_{3}I,\\ \frac{dE}{dt} & =\left(1‒p\right)\left[\left(1‒\varphi \right)\lambda_{1}S_{1}I+\lambda_{2}S_{2}I+\lambda_{3}S_{3}I\right]‒vE‒dE,\\ \frac{dI}{dt} & =p\left[\left(1‒\varphi \right)\lambda_{1}S_{1}I+\lambda_{2}S_{2}I+\lambda_{3}S_{3}I\right]‒\left[d+\left(1+\xi \right)\gamma +\mu \right]I+vE+\eta R,\\ \frac{dR}{dt} & =\left(1+\xi \right)\gamma I‒(d+\eta )R. \end{array}\right.$$

### 2.3. Theoretical Results of Model (2)

In epidemiology, the basic reproduction number (denoted $R_{0}$) of an infection can be viewed as the number of cases one case generates on average over the course of its infectious period [28]. This is one of the most important indexes in evaluating the risk of an infectious disease. The asymptotical dynamic behavior of infectious diseases can be reflected by the steady state, which implies the disease will die out or persist in the future. Therefore, we first provided some mathematical analysis results of Model (2), whose proofs are shown in Appendix A.

- Model (2) has the following positively invariant set:
  (3) $$\begin{array}{c} \Omega =\left\{\left(S_{1},S_{2},S_{3},E,I,R\right)|S_{1},S_{2},S_{3},E,I,R\geq 0,0\leq S_{1}+S_{2}+S_{3}+E+I+R\leq \frac{A}{\hat{d}}\right\}. \end{array}$$
- Making use of the next generation matrix (see [29]), we obtained the basic reproduction number of Model (2) as follows:
  (4) $$R_{0}=\frac{\left(\nu +pd\right)[\left(1‒\varphi \right)\lambda_{1}S_{1}^{0}+\lambda_{2}S_{2}^{0}+\lambda_{3}S_{3}^{0}]}{\left(\nu +d)[d+(1+\xi )\gamma +\mu \right]}\left[1+\frac{\eta (1+\xi )\gamma }{\left(d+\eta )[d+(1+\xi )\gamma +\mu \right]}\right].$$
- This model has a disease-free equilibrium $P^{0}=\left(S_{1}^{0},S_{2}^{0},S_{3}^{0},0,0,0\right)$, where
  (5) $$S_{1}^{0}=\frac{A}{d_{1}+m_{1}},\;S_{2}^{0}=\frac{m_{1}A}{\left(d_{1}+m_{1}\right)\left(d_{2}+m_{2}\right)},\;S_{3}^{0}=\frac{m_{1}m_{2}A}{\left(d_{1}+m_{1}\right)\left(d_{2}+m_{2}\right)d_{3}},$$
  and the endemic equilibrium $P^{*}=\left(S_{1}^{*},S_{2}^{*},S_{3}^{*},E^{*},I^{*},R^{*}\right)$, which is determined by the following equations
  (6) $$\left\{\begin{array}{c} A‒d_{1}S_{1}‒m_{1}S_{1}‒\lambda_{1}S_{1}I=0,\\ m_{1}S_{1}‒d_{2}S_{2}‒\lambda_{2}S_{2}I‒m_{2}S_{2}=0,\\ m_{2}S_{2}‒d_{3}S_{3}‒\lambda_{3}S_{3}I=0,\\ \left(1‒p\right)[\left(1‒\varphi \right)\lambda_{1}S_{1}I+\lambda_{2}S_{2}I+\lambda_{3}S_{3}I]‒vE‒dE=0,\\ p\left[\left(1‒\varphi \right)\lambda_{1}S_{1}I+\lambda_{2}S_{2}I+\lambda_{3}S_{3}I\right]‒\left[d+\left(1+\xi \right)\gamma +\mu \right]I+vE+\eta R=0,\\ \gamma I‒dR‒\eta R=0. \end{array}\right.$$
- If $R_{0}<1$, the disease-free equilibrium $P^{0}$ is globally asymptotically stable.

## 3. Numerical Simulations and Sensitivity Analysis

Despite the central government completing two 10-year control plans, many difficulties still exist elsewhere in the country’s TB control programs. The spread of severe acute respiratory syndrome (SARS) in 2003 revealed substantial weaknesses in the country’s public health system. After the SARS epidemic was controlled, the government made better efforts to tackle public health problems, and increased public health funding, revised laws that concerned the control of infectious diseases, implemented the world’s largest Internet-based disease reporting system, and started a program to rebuild local public health facilities. These measures contributed to an acceleration in the efforts to control tuberculosis [30,31]. Because the data quality for TB is higher after 2004, we decided to fit the data for the infected TB cases for 2005–2016 in China using Model (2). The data from, 2005–2015 were used to fit and those of 2016 were used to check the predictive power by residual and $R^{2}$ statistic.

### 3.1. Parameter Estimation

To perform the numerical simulations, we first needed to estimate the model parameters. According to the existing literature and related results of the Chinese population statistic yearbook, we estimated the parameters. The values of the parameters are listed in Table 2, and the detailed estimation process of the parameter values are as follows.

**Figure 3:**
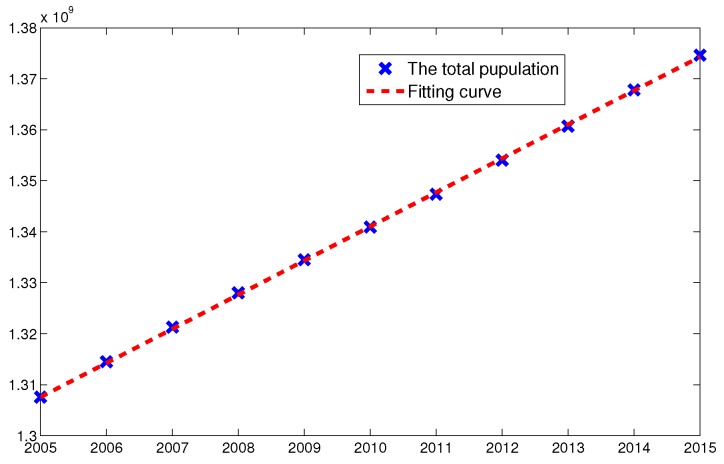
The fitting curve of parameters $m_{1}$ and $m_{2}$ by L-S method using the total population from 2005 to 2015. The blue cross point and red dashed line represent the total number of population and the fitted curve, respectively.

(a) From the results in the China population statistic yearbook from 2005 to 2015 [32], we obtained the natural death rate of the entire population and those of the three age groups. Hence, the mean and the 95% confidence interval are d≈0.0067, 95% CI (0.0065,0.0069), $d_{1}\approx 0.0017$, 95% CI (0.0013,0.0021), $d_{2}\approx 0.0023$, 95% CI (0.0023,0.0024), $d_{3}\approx 0.0367$, 95% CI (0.0352,0.0382) and $A\approx 1.623\times 10^{7}$, 95% CI $\left(1.606\times 10^{7},1.643\times 10^{7}\right)$.

(b) Based on the proportions of age groups in 2005, we calculated the initial value $S_{1}\left(0\right)=26,504\times 10^{4},S_{2}\left(0\right)=94,197\times 10^{4}$, $S_{3}\left(0\right)=10,055\times 10^{4}$. The initial number of people infected with TB is I(0)=1,259,308, which is the number of people infected with TB in 2005. Moreover, the percent ages of people with a TB bacteria infection but asymptomatic and those successfully treated for TB are 12.1% and 80%, respectively [3]. In 2004, the number of people infected with TB was 970,279, so we assumed the initial value of E(0)≈970,279×12.1%=117,403 and R(0)≈970,279×80%=776,223.

(c) Using the following system

(7) $$\left\{\begin{array}{cc} \frac{dS_{1}}{dt} & =A‒d_{1}S_{1}‒m_{1}S_{1},\\ \frac{dS_{2}}{dt} & =m_{1}S_{1}‒d_{2}S_{2}‒m_{2}S_{2},\\ \frac{dS_{3}}{dt} & =m_{2}S_{2}‒d_{3}S_{3}, \end{array}\right.$$

and the census data of total population in China from 2005 to 2015, we estimated the parameters $m_{1}$ and $m_{2}$ by nonlinear Least-Square method (see Figure 3).

(d) The latent period of TB is about two months [33], thus we calculated the re-activation rate of latent TB patients $v=\frac{12}{2}=6$ annually. From the 2013 WHO global tuberculosis report [2], we obtained the disease-induced death rate μ=0.0025, and from Blower et al. [10], we knew the fraction of fast-developing infectious cases *p* is 0.05 and the recovery rate is γ=0.496. According to the Fifth national TB epidemiological survey [3], we knew that the incremental recovery rate of TB ξ is 0.51 and φ is 0.9.

(e) By use of Model (2), we simulated the cumulative number of people infected with TB from 2005 to 2016. The infection rate values $\lambda_{1},\lambda_{2},\lambda_{3}$ were obtained by the nonlinear Least-Square method. First, we let X(t) denote the cumulative number of people infected with TB at time *t*. According to the flow chart of TB transmission by age grouping (Figure 2), we knew that three parts contributed to the number of infectious compartments: the number of infected people from the three susceptible age group , the latency, and the TB recurrence from recovery:

(8) $$\frac{dX(t)}{dt}=p\left[\left(1‒\varphi \right)\lambda_{1}S_{1}I+\lambda_{2}S_{2}I+\lambda_{3}S_{3}I\right]+vE+\eta R.$$

where X(t) represents the cumulative number of people infected with TB at time *t*, and I(t) denotes the number in compartment *I* at time *t*, which includes the newly-infected TB cases and recovery TB cases at time *t*. Thus, to estimate the newly-infected TB cases, we had Z(t)=X(t)‒X(t‒1) represent the newly-infected TB cases. In the following, we used Z(t) to simulate the reported TB infected cases per year.

### 3.2. Numerical Simulations from 2005 to 2016

The decrease in infected TB cases may be due to the current control strategies not being fully effective [31], which aligns with the dynamic behaviors of Model (2). China developed and implemented two five-year national plans in the 1980s and one 10-year national plan in the 1990s to control TB. After implementing these national TB control programs, the full modern TB control strategy was implemented. The increase of TB in China has since been effectively controlled.

**Figure 4:**
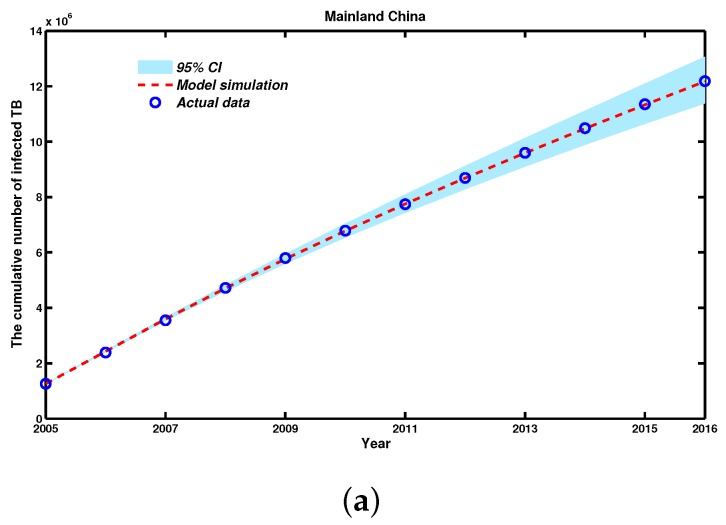

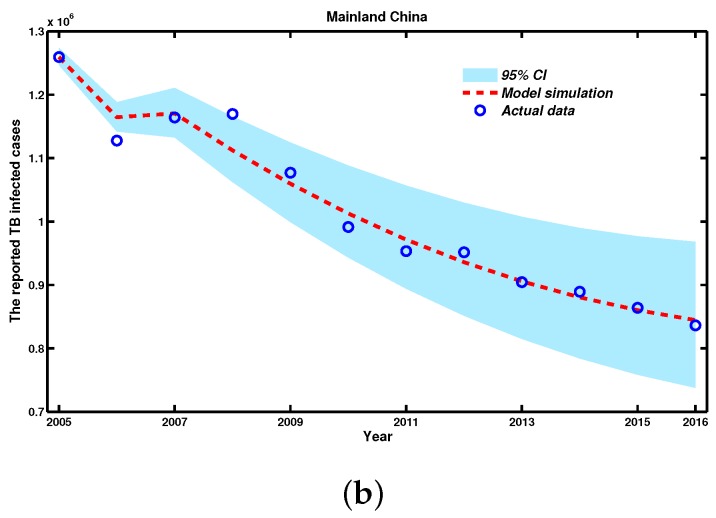
The fitting curve and observed data from 2005 to 2016 in Mainland China with Model (2). (**a**) The simulation result for the cumulative number of people infected with TB; (**b**) The simulation result for newly-infected TB cases, where the red dashed line is the simulated curve of Model (2), the light grey area is the 95% confidence interval (CI) for all 2000 simulations, and the blue circles are the reported TB infection cases.

With help from the MATLAB (The Mathworks, Inc., Natick, MA, USA) tool fminsearch, which is part of the optimization toolbox, we estimated the optimal parameters for Model (2). Then, using the fourth-order and five-order Runge-Kutta algorithm (ode45 function), which is a powerful tool for solving ordinary differential equations, according to the corresponding parameters of Model (2) listed in Table 2, we simulated the data of the cumulative number and reported cases of TB infection from 2005 to 2016. Meanwhile, by random sampling of the 95% confidence interval (CI) of the parameters, we further plotted the 95% CI of the trajectories of the TB infection data, both cumulative and newly-infected TB cases, based on 2000 independent repeated simulations of Model (2) (see Figure 4).

Figure 4 shows both the time evolution of infection cases and a comparison with the empirical records of TB infection cases, and also shows the 95% percent interval for all 3000 passing simulation trajectories. Moreover, we calculated the residual of 2016 as 235 and R-square (R${}^{2}$) statistic to show goodness of fit [34], where the R-square value is 0.9812. We also observed that the actual reported TB infection data almost fell into the 95% CI of our simulation trajectories. Thus, our simulation results are in good accordance with the reported TB infection data, both cumulative and newly-infected TB cases, from the CDC in China from 2005 to 2016. Model (2) had a better predictive performance.

**Figure 5:**
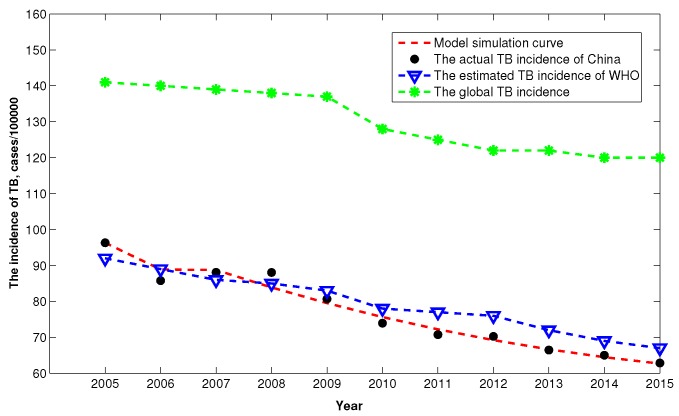
The comparison of TB incidence from 2005 to 2015.

In addition, to evaluate the TB burden of China based on our model, according to the definition of incidence that the number of new and relapse cases of TB arising in a given time period, usually one year, we can further translate the reported infected TB cases into the incidence rate of TB. For comparison, we also plot the global TB incidence and the estimated TB incidence of WHO from 2005 to 2015. Figure 5 shows that after 2008, the TB incidence is lower than that of estimated value by WHO, which may implies that China substantial accelerate the control effects of TB. Moreover, we can observed that the TB incidence of China is far below the global level.

### 3.3. Uncertainty and Sensitivity Analysis of $R_{0}$

**Figure 6:**
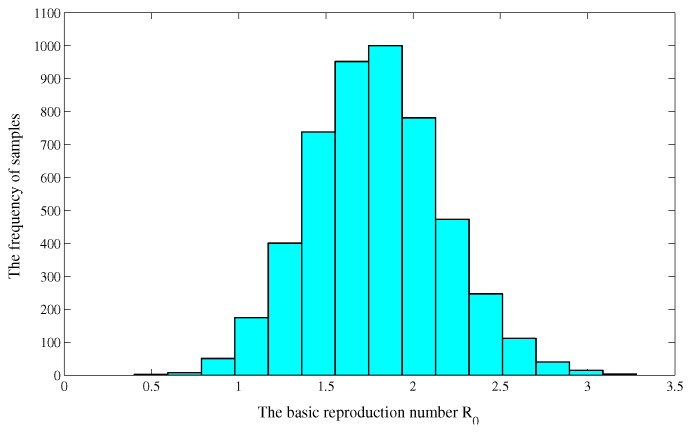
The numerical distribution of the basic reproduction number $R_{0}$.

Due to the uncertainty in the initial parameter estimates, we performed a Latin hypercube sampling (LHS) on the estimated parameters (see, e.g., [8,35,36]). Since the LHS requires assigning a probability density function (PDF) to each of the parameters, we stratified the PDFs into 3000 equiprobability areas and then independently randomly sampled 3000 times without replacement, forming 3000 input parameter vectors [21].These input parameter vectors were then used to calculate the numerical distribution of the basic reproduction number $R_{0}$. With the simulated parameter values, we obtained the numerical distribution of the basic reproduction number $R_{0}$ (see Figure 6), and estimated the basic reproduction number from 2005 to 2016 is $R_{0}=1.7858$ and the 95% confidence interval of $R_{0}$ is (1.7752,1.7963).

For the sensitivity analysis of $R_{0}$, we can calculate partial rank correlation coefficient (PRCC), which reflects the correlation between parameters $A,\lambda_{1},\lambda_{2},\lambda_{3},m_{1},m_{2},\gamma ,\eta$ and $R_{0}$. The PRCC of the estimated parameters with respect to $R_{0}$ are listed in Table 3. It follows from Table 3 that there exist a positive correlation between $A,\lambda_{1},\lambda_{2},\lambda_{3},m_{1},\eta$ and $R_{0}$, and a negative correlation between $m_{2},\gamma$ and $R_{0}$. Furthermore, we can obtain that $|PRCC\left(A\right)|>|PRCC\left(\gamma \right)|>|PRCC\left(\lambda_{3}\right)|>|PRCC\left(m_{2},\lambda_{1},\lambda_{2},\eta ,m_{1}\right)|$, namely, $A,\gamma ,\lambda_{3}$ play the most important role to determine $R_{0}$.

### 3.4. Feasibility Assessment of Reaching WHO End TB Strategy

Significant progress in controlling TB has been made during the last two decades, however, the WHO proposed a post-2015 global End TB Strategy in 2014 [37]. This strategy aims to end the global TB epidemic, with targets to cut new cases by 90% by 2035 and a milestones of 50% reduction in TB incidence rate in 2025.

**Figure 7:**
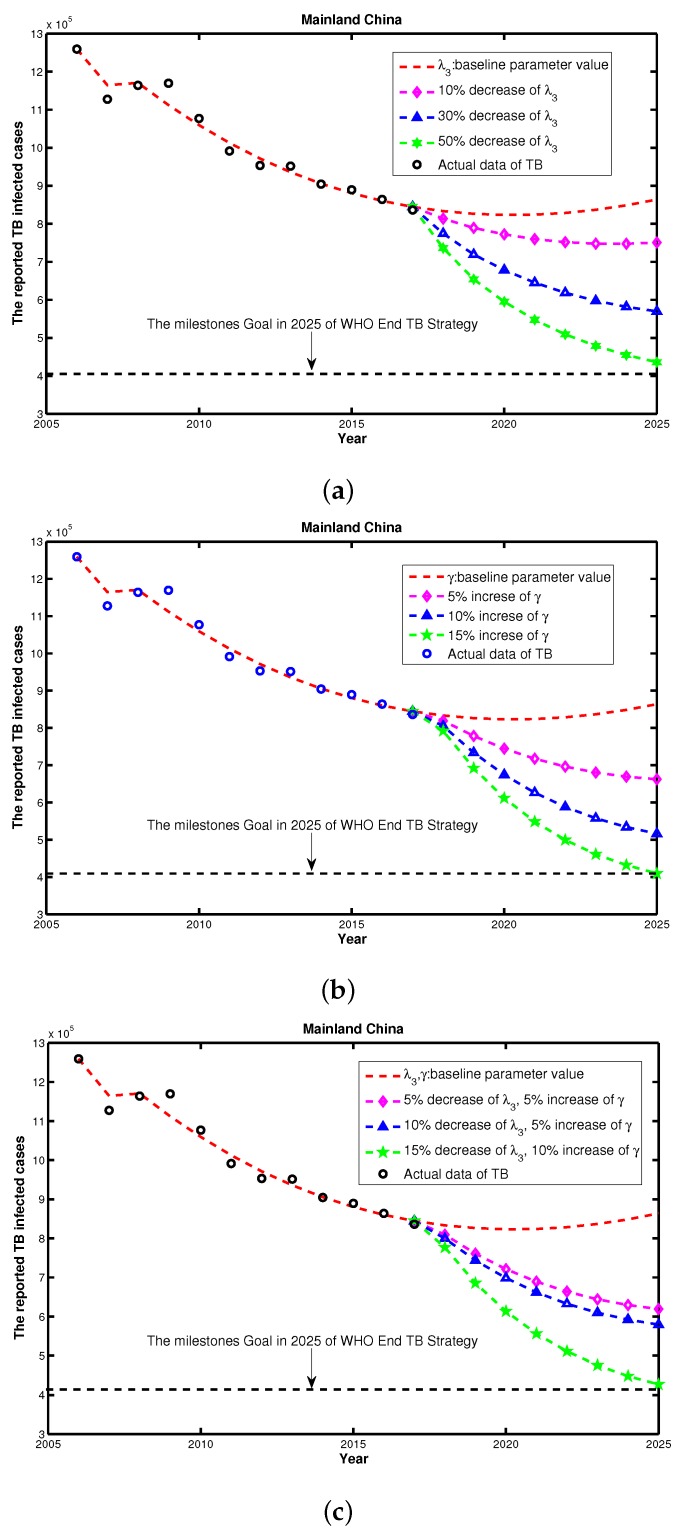
The reported TB cases (**a**) under control strategies of decreasing $\lambda_{3}$ from 1 to 0.5; (**b**) under control strategies of increasing γ from 1 to 1.15; and (**c**) under a combination of control strategies of increasing γ from 1 to 1.1 and decreasing $\lambda_{3}$ from 1 to 0.85, simultaneously.

In the above analysis, γ and $\lambda_{3}$ are the most important risk factors for TB control. To examine the TB controlling effects with respect to γ and $\lambda_{3}$, we examined if reaching the WHO End TB Strategy would be feasible based on the current different control strategies. We used the parameter values listed in Table 2 as a baseline to compare the control effects. First, we only considered the single intervention scenario including $\lambda_{3}$, and, as shown in Figure 7a, we would not be able to reach the goal of WHO End TB Strategy under the current plan, even with decreasing $\lambda_{3}$ by 50%. Then, we considered the single intervention scenario of γ, and as shown in Figure 7b, 15% increasing of the baseline γ would allow us to reach the WHO target. Finally, we considered an integrated control strategy including both γ and $\lambda_{3}$ simultaneously. Figure 7c shows that if we can reduce the morbidity in the senior group $\lambda_{3}$ by 15%, and increase the recovery rate γ by 10%, then we will meet the TB End Target. Therefore, we concluded that, by using the current TB control interventions, China may not reach the WHO End TB Strategy in 2025. To achieve the WHO End TB Strategy goal, China will need to pay more attention to enhance their combination TB interventions and further explore the feasibility of additional control strategies.

## 4. Discussion

The Millennium Development Goal’s target in China was achieved with the decrease in the reported number of TB cases, however, the aging demographic represents an increasing challenge to TB control as China considers its post-2015 End TB Strategy [24]. Importantly, significant differences exist among different age groups in terms of the morbidity of TB. Taking this into account, and using the reported TB data in China from 2005 to 2016, we proposed a SEIR epidemic model with three age groups, children, middle-aged, and senior, to study the transmission of tuberculosis in China. By means of the Least Square method, we evaluated the parameters and simulated the model, and the model agrees well with the annual reported TB data in China. Furthermore, we calculated the basic reproduction number $R_{0}\approx 1.7858$, and obtain the 95% confidence interval for $R_{0}$ is about (1.7752,1.7963) by Latin hypercube sampling. We also assessed the feasibility of reaching the WHO End TB Strategy goal under current China TB control initiatives by using a sensitivity analysis of $R_{0}$ in terms of the parameters.

(i) Our results demonstrate that taking the age grouping into consideration is reasonable to characterize the transmission and to improve the control strategies of targeting therapy for TB in China. Based on the age-structuring model, more risk factors for different age groups can be identified. Interventions could be targeted toward specific groups, which would be particularly effective as an epidemic control measure [38]. Thus, the age grouping pattern provides a meaningful scheme, based upon the treatment of active cases and the chemoprophylaxis of latently infected individuals, to define targeted sub-populations for treating TB infections. For instance, the BCG vaccine is useful only for younger people but is less effective for the middle- or the senior-aged groups, having an average efficacy of only about 50% for those groups [2,39]. However, with the aging of the Chinese population and high morbidity rate of TB in seniors, perhaps an analogue of the BCG vaccine control strategy should be implemented for the potentially high-risk senior sub-population, which may result in the decreasing the morbidity in that group. In addition, the nationwide DOTS program should be more focused on the senior-aged group, such as providing more financial assistance for this group, who may experience catastrophic costs due to TB [26], and should place more emphasis on the people with latent TB in middle-aged group, who may increase the proportion of the actively infected people in the senior group.

(ii) From the analysis of PRCC of $R_{0}$ in Table 3, it is shown that γ, $\lambda_{3}$, $m_{2}$ and $\lambda_{1}$ are the most effective methods for controlling TB in China. Although the WHO’s target treatment levels may not lead to eradication, these non-eradication treatment levels could significantly reduce morbidity and mortality [11]. Thus, two important indexes must be improved: First, the TB treatment success rate and treatment coverage (increasing γ), for example, by providing high-quality TB care to prevent suffering and death from TB. Second, monitoring and detecting the latent TB in the senior population (reducing $\lambda_{3}$) may help prevent the development of active TB in those already infected with Mycobacterium tuberculosis, including further strengthening the public health facilities and providing an isolation policy for those with detected latent TB. For TB infection in children, contact tracing is one of the key components of TB prevention, so educational programming and campaigning can be aimed the youngest age group.

(iii) Our feasibility assessment of reaching WHO End TB Strategy goal for 2015–2025, showed that even with any single intervention or combination of interventions, China may not reach the goal at the country level, as shown by the multi-models result in Houben et al. [5]. Due to the influence of drug-resistant strains, co-infection with other diseases including HIV, diabetes mellitus, etc., and increasing infection opportunities that accompanies world travel, TB will be weakly persistent and should show an overall decreasing trend in the future (see Figure 7). shows that if we can reduce the morbidity of the senior group by 15%, and increase the recovery rate by 10% , then we could potentially achieve the WHO TB End Target. Similar to Wang et al. [31] pointed out, China is not on track, nor does it appear to be currently possible, to reach the required reduction in prevalence. Therefore, there is still a need for sustained improvements in TB control to keep reducing the burden of TB in China.

From a practical viewpoint, clarifying the role of age in the transmission of TB may aid in forecasting the long-term health risks, in proposing a targeted TB control strategy, and in setting objectives and using limited resources more effectively [19].

## Figures and Tables

**Table 1:** Reported infected TB cases from 2005 to 2016 in China.

Year	2005	2006	2007	2008	2009	2010
Cases	1,259,308	1,127,571	1,163,959	1,169,540	953,275	951,508
Year	2011	2012	2013	2014	2015	2016
Cases	1,076,938	991,350	889,381	864,015	864,015	836,236

**Table 2:** The parameter values of Model (2).

Parameter	Value	Confidence Interval	Source
*A*	$1.623\times 10^{7}$	$\left(1.606\times 10^{7},1.643\times 10^{7}\right)$	[32]
*d*	0.007	(0.00688, 0.00702)	[32]
$d_{1}$	0.0017	(0.0013, 0.0021)	[32]
$d_{2}$	0.0023	(0.0023, 0.0024)	[32]
$d_{3}$	0.0367	(0.0352,0.0382)	[32]
$m_{1}$	0.079	−	Fitting
$m_{2}$	0.0067	−	Fitting
μ	0.0025	−	[2]
*p*	0.05	(0,0.3)	[10]
γ	0.496	−	[10]
ξ	0.51	−	[3]
*v*	6	−	[33]
η	0.00341	−	[30]
φ	0.9	−	[3]
$\lambda_{1}$	$1.268\times 10^{‒10}$	−	Fitting
$\lambda_{2}$	$5.11\times 10^{‒10}$	−	Fitting
$\lambda_{3}$	$2.553\times 10^{‒9}$	−	Fitting

**Table 3:** The PRCC of the estimated parameters with respect to $R_{0}$.

Parameters	PRCC	*p*-Value
*A*	0.9961	0.0005
γ	−0.7482	0.0009
$\lambda_{3}$	0.6141	0.0017
$m_{2}$	−0.5265	0.0001
$\lambda_{1}$	0.5195	0.004
$\lambda_{2}$	0.4820	0.0022
η	0.1808	0.0036
$m_{1}$	0.0175	0.0012

## Appendix A

### Appendix A.1. Proof of the Positively Invariant Set

Adding the first five equations in Model (2), we have

$$\begin{array}{ccc} \frac{d(S_{1}+S_{2}+S_{3}+E+I+R)}{dt} & = & A‒d_{1}S_{1}‒d_{2}S_{2}‒d_{3}S_{3}‒dE‒\varphi \lambda_{1}S_{1}I‒\left(d+\mu \right)I‒dR\\ & \leq & A‒\hat{d}\left(S_{1}+S_{2}+S_{3}+E+I+R\right), \end{array}$$

where $\hat{d}=\min\left\{d_{1},d_{2},d_{3},d,d+\mu \right\}$. It follows from the above equation that

(A1) $$\underset{t\to \infty }{lim sup}\left(S_{1}+S_{2}+S_{3}+E+I+R\right)\leq \frac{A}{\hat{d}}.$$

Thus, we can obtain the following positively invariant set of Model (2):

$$\begin{array}{c} \Omega =\left\{\left(S_{1},S_{2},S_{3},E,I,R\right)|S_{1},S_{2},S_{3},E,I,R\geq 0,0\leq S_{1}+S_{2}+S_{3}+E+I+R\leq \frac{A}{\hat{d}}\right\}. \end{array}$$

### Appendix A.2. Proof of the Existence of Positive Steady State

By a simple calculation, we have from (6) that

(A2) $$S_{1}=\frac{A}{d_{1}+m_{1}+\lambda_{1}I},\;S_{2}=\frac{m_{1}A}{\left(d_{1}+m_{1}+\lambda_{1}I\right)\left(d_{2}+m_{2}+\lambda_{2}I\right)},\;S_{3}=\frac{m_{1}m_{2}A}{\left(d_{1}+m_{1}+\lambda_{1}I\right)\left(d_{2}+m_{2}+\lambda_{2}I\right)\left(d_{3}+\lambda_{3}I\right)},\;E=\frac{\left(1‒p\right)[\left(1‒\varphi \right)\lambda_{1}S_{1}I+\lambda_{2}S_{2}I+\lambda_{3}S_{3}I]}{v+d},\;R=\frac{\gamma I}{d+\eta }.$$

Substituting (A2) into the fifth equation in (6) yields

(A3) $$f\left(I\right)=\left(1‒\varphi \right)\lambda_{1}S_{1}+\lambda_{2}S_{2}+\lambda_{3}S_{3}‒\frac{\left(1‒\varphi \right)\lambda_{1}S_{1}^{0}+\lambda_{2}S_{2}^{0}+\lambda_{3}S_{3}^{0}}{R_{0}}=0,$$

We can see from the expressions of $S_{1},S_{2}$ and $S_{3}$ in (A2) that

$$\frac{dS_{1}}{dI}<0,\;\;\frac{dS_{2}}{dI}<0,\;\;\frac{dS_{3}}{dI}<0.$$

It follows from (A3) that f(I) is monotonously decreasing with respect to *I* in $\left[0,\frac{A}{\hat{d}}\right]$. If $R_{0}>1$, we can compute that $f\left(0\right)=\left(1‒\varphi \right)\lambda_{1}S_{1}^{0}+\lambda_{2}S_{2}^{0}+\lambda_{3}S_{3}^{0}‒\frac{\left(1‒\varphi \right)\lambda_{1}S_{1}^{0}+\lambda_{2}S_{2}^{0}+\lambda_{3}S_{3}^{0}}{R_{0}}>0$ and $f(\frac{A}{\hat{d}})<0$. Thus Equation (A3) has a unique positive solution $I^{*}\in \left(0,\frac{A}{\hat{d}}\right)$. Submitting $I=I^{*}$ into (7) we obtain the unique endemic equilibrium $P^{*}=\left(S_{1}^{*},S_{2}^{*},S_{3}^{*},E^{*},I^{*},R^{*}\right)$ of model (2).

### Appendix A.3. Proof of the Global Stability of P^0^

First, let us define a Lyapunov function $L=\frac{\nu }{\nu +d}E+I+\frac{\eta }{d+\eta }R$, we can get

$$\begin{array}{cc} \frac{dL}{dt} & =\frac{\nu }{\nu +d}\left[\left(1‒p\right)(\left(1‒\varphi \right)\lambda_{1}S_{1}I+\lambda_{2}S_{2}I+\lambda_{3}S_{3}I)‒\left(\nu +d\right)E\right]\\ & \;+p\left(\left(1‒\varphi \right)\lambda_{1}S_{1}I+\lambda_{2}S_{2}I+\lambda_{3}S_{3}I\right)‒\left(d+\left(1+\xi \right)\gamma +\mu \right)I+\nu E+\eta R+\frac{\eta (1+\xi )\gamma }{d+\eta }I‒\eta R\\ & =\left\{\left[\frac{\nu }{\nu +d}\left(1‒p\right)+p\right]\left(\left(1‒\varphi \right)\lambda_{1}S_{1}+\lambda_{2}S_{2}+\lambda_{3}S_{3}\right)‒\left(d+\left(1+\xi \right)\gamma +\mu \right)\right\}I‒\frac{\eta (1+\xi )\gamma }{d+\eta }I\\ & =\frac{1}{\left[d+(1+\xi )\gamma +\mu \right]}\left\{\frac{\nu +pd}{\nu +d}\frac{\left(1‒\varphi \right)\lambda_{1}S_{1}+\lambda_{2}S_{2}+\lambda_{3}S_{3}}{d+(1+\xi )\gamma +\mu }‒1‒\frac{\eta (1+\xi )\gamma }{\left(d+\eta )[d+(1+\xi )\gamma +\mu \right]}\right\}I\\ & \leq \frac{1}{\left[d+(1+\xi )\gamma +\mu \right]}\left(R_{0}‒1\right)I. \end{array}$$

When $R_{0}<1$, we know that $\frac{dL}{dt}=0$ if and only if $S_{1}=S_{1}^{0},S_{2}=S_{2}^{0},S_{3}=S_{3}^{0}$, otherwise $\frac{dL}{dt}<0$. Thus, by LaSalle’s invariant set principle [40], it follows that the disease-free equilibrium $P^{0}$ is globally asymptotically stable in the set Ω when $R_{0}<1$.  ☐

## References

[1] Blower S.M., Daley C.L. (2002). Problems and solutions for the stop tb partnership. Lancet Infect. Dis..

[2] World Health Organization (2013). Global Tuberculosis Report.

[3] Wang Y. (2012). The fifth national tuberculosis epidemiological survey in 2010. Chin. J. Autituberc..

[4] Smith J.P., Strauss S., Zhao Y.H. (2014). Healthy Aging in China. J. Econ. Ageing.

[5] Houben M.G., Wu C.Y., Rhines A.S., Denholm J.T., Gomez G.B., Hippner P. (2016). Feasibility of achieving the 2025 WHO global tuberculosis target in South Africa, China, and India: A combined analysis of 11 mathematical models. Lancet Glob. Health.

[6] Li M.T., Sun G.Q., Wu Y.F., Zhang J., Jin Z. (2014). Transmission dynamics of a multi-group brucellosis model with mixed cross infection in public farm. Appl. Math. Comput..

[7] Waaler H.T., Gese A., Anderson S. (1962). The use of mathematical models in the study of the epidemiology of tuberculosis. Am. J. Public Health.

[8] Li M.T., Sun G.Q., Zhang J., Jin Z., Sun X.D., Wang Y.M., Huang B.X., Zheng Y.H. (2014). Transmission dynamics and control for a brucellosis model in hinggan league of inner mongolia, China. Math. Biosci. Eng..

[9] Li M.T., Jin Z., Sun G.Q., Zhang J. (2014). Modeling direct and indirect disease transmission using multi-group model. J. Math. Anal. Appl..

[10] Blower S.M., McLean A.R., Porco T.C. (1995). The intrinsic transmission dynamics of tuberculosis epidemics. Nat. Med..

[11] Blower S.M., Small P.M., Hopewell P.C. (1996). Control strategies for tuberculosis epidemics: New models for old problems. Science.

[12] Dye C., Watt C.J., Bleed D. (2002). Low access to a high-cure therapy: A challenge for international tuberculosis control. Bull. World Health Organ..

[13] Jia Z.W., Tang G.Y., Jin Z. (2008). Modeling the impact of immigration on the epidemiology of tuberculosis. Theor. Popul. Biol..

[14] Bhunu C.P., Garira W., Mukandavire Z., Zimba M. (2008). Tuberculosis transmission model with chemoprophylaxis and treatment. Bull. Math. Biol..

[15] Bowong S., Kurths J. (2012). Modeling and analysis of the transmission dynamics of tuberculosis without and with seasonality. Nonlinear Dyn..

[16] Cao H., Zhou Y.C. (2012). The discrete age-structured SEIT model with application to tuberculosis transmission in China. Math. Comput. Model..

[17] Feng Z.L., Castillo C.C., Capurro A.F. (2000). A model for tuberculosis with exogenous reinfection. Theor. Popul. Biol..

[18] Kar T.K., Mondal P.K. (2012). Global dynamics of a tuberculosis epidemic model and the influence of backward bifurcation. J. Math. Model. Algor..

[19] Liu L.J., Zhao X.Q., Zhou Y.C. (2010). A tuberculosis model with seasonality. Bull. Math. Biol..

[20] Zhou X.Y., Shi X.Y., Cheng H.D. (2013). Modelling and stability analysis for a tuberculosis model with healthy education and treatment. Comput. Appl. Math..

[21] Whang S., Choi S., Jung E. (2011). A dynamic model for tuberculosis transmission and optimal treatment strategies in South Korea. J. Theor. Biol..

[22] Okuonghae D., Omosigho S.E. (2011). Analysis of a mathematical model for tuberculosis: What could be done to increase case detection. J. Theor. Biol..

[23] Mccluskey C.C., Driessche P.V. (2004). Global analysis of two tuberculosis models. J. Dyn. Diff. Equ..

[24] Huynh G.H., Klein D.J., Chin D.P., Wagner B.G., Eckhoff P.A., Liu R.Z., Wang L.X. (2015). Tuberculosis control strategies to reach the 2035 global targets in China: The role of changing demographics and reactivation dsease. BMC Med..

[25] White P.J., Garnett G.P. (2010). Mathematical modelling of the epidemiology of tuberculosis. Modeling Parasite Transformation and Control 673. Adv. Exp. Med. Biol..

[26] World Health Organization (2017). Tuberculosis. http://www.who.int/mediacentre/factsheets/fs104/en/.

[27] Lowrie D.B. (2012). Tuberculosis vaccine research in China. Emerg. Microbes Infect..

[28] Fraser C., Donnelly C.A., Cauchemez S., Hanage W.P., Van Kerkhove M.D., Hollingsworth T.D., Griffin J., Baggaley R.F., Jenkins H.E., Lyons E.J. (2009). Pandemic Potential of a Strain of Influenza A (H1N1): Early Findings. Science.

[29] Van den Driessche P., Watmough J. (2002). Reproduction numbers and sub-threshold endemic equilibria for compartmental models of disease transmission. Math. Biosci..

[30] Millet J.P., Shaw E., Orcau A., Casals M., Miro J.M., Cayla J.A. (2013). The Barcelona Tuberculosis Recurrence Working Group’ Tuberculosis Recurrence after Completion Treatment in a European City: Reinfection or Relapse?. PLoS ONE.

[31] Wang L.D., Liu J.J., Chin D.P. (2007). Progress in tuberculosis control and the evolving public-health system in china. Lancet.

[32] (2017). China Population Statistic Yearbook. http://www.stats.gov.cn/tjsj/ndsj/.

[33] (2017). National Scientific Data Sharing Platform for Population and Health. http://www.ncmi.cn/info/69/1544.

[34] Devore J.L. (2011). Probability and Statistics for Engineering and the Sciences.

[35] Blower S.M., Dowlatabadi H. (1994). Sensitivity and uncertainty analysis of complex models of disease transmission: An HIV model as an example. Int. Stat. Rev..

[36] Sanchez M.A., Blower S.M. (1997). Uncertainty and sensitivity analysis of the basic reproductive rate: Tuberculosis as an example. Am. J. Epidemiol..

[37] World Health Organization (2014). WHO End TB Strategy. http://www.who.int/tb/post2015$_$strategy/en/.

[38] Ziv E., Daley C.L., Blower S.M. (2001). Early therapy for latent tuberculosis infection. Am. J. Epidemiol..

[39] Ziv E., Daley C.L., Blower S.M. (2004). Potential public health impact of new tuberculosis vaccines. Emerg. Infect. Dis..

[40] LaSalle J.P. (1976). The Stability of Dynamical Systems, Regional Conference Series in Applied Mathematics.

